# Randomized Controlled Trial of Hospital-Based Hygiene and Water Treatment Intervention (CHoBI7) to Reduce Cholera

**DOI:** 10.3201/eid2202.151175

**Published:** 2016-02

**Authors:** Christine Marie George, Shirajum Monira, David A. Sack, Mahamud-ur Rashid, K.M. Saif-Ur-Rahman, Toslim Mahmud, Zillur Rahman, Munshi Mustafiz, Sazzadul Islam Bhuyian, Peter J. Winch, Elli Leontsini, Jamie Perin, Farzana Begum, Fatema Zohura, Shwapon Biswas, Tahmina Parvin, Xiaotong Zhang, Danielle Jung, R. Bradley Sack, Munirul Alam

**Affiliations:** Johns Hopkins University, Baltimore, Maryland, USA (C.M. George, D.A. Sack, P.J. Winch, E. Leontsini, J. Perin, X. Zhang, D. Jung, R.B. Sack);; icddr,b, Dhaka, Bangladesh (S. Monira, M. Rashid, K.M. Saif-Ur-Rahman, T. Mahmud, Z. Rahman, M. Mustafiz, S.I. Bhuyian, F. Begum, F. Zohura, S. Biswas, T. Parvin, M. Alam)

**Keywords:** cholera, hand washing, water treatment, household contacts, enteric infections, randomized controlled trial, bacteria, Bangladesh, prevention

## Abstract

The risk for cholera infection is >100 times higher for household contacts of cholera patients during the week after the index patient seeks hospital care than it is for the general population. To initiate a standard of care for this high-risk population, we developed Cholera-Hospital-Based-Intervention-for-7-Days (CHoBI7), which promotes hand washing with soap and treatment of water. To test CHoBI7, we conducted a randomized controlled trial among 219 intervention household contacts of 82 cholera patients and 220 control contacts of 83 cholera patients in Dhaka, Bangladesh, during 2013–2014. Intervention contacts had significantly fewer symptomatic *Vibrio cholerae* infections than did control contacts and 47% fewer overall *V. cholerae* infections. Intervention households had no stored drinking water with *V. cholerae* and 14 times higher odds of hand washing with soap at key events during structured observation on surveillance days 5, 6, or 7. CHoBI7 presents a promising approach for controlling cholera among highly susceptible household contacts of cholera patients.

## Abstract 2

This intervention significantly reduced symptomatic *Vibrio cholerae* infection.

Severe cholera without adequate rehydration kills up to half of affected persons (*1*). The World Health Organization estimates that 3–5 million cholera cases occur worldwide each year (*2*). Studies have identified multiple risk factors for *Vibrio cholerae* infection, such as drinking street-vended water, placing one’s hands into stored household water, lack of drinking water treatment, eating food prepared by a recently ill food handler, and not washing hands with soap before eating food (*3*–*8*). These findings suggest that cholera is transmitted through contaminated water and poor hygiene practices. Therefore, interventions targeting improved water treatment and storage practices and hand washing with soap have the potential to substantially reduce cholera transmission (*8*).

Previous studies in Bangladesh have demonstrated that household contacts of cholera patients are at >100 times higher risk for cholera infections during the 1-week period after the index patient seeks hospital care (*5*,*7*,*9*–*11*). Although the average rate of cholera in National Institute of Health–sponsored surveillance areas of Bangladesh is 1.6 cases/1,000 persons, a study in urban Dhaka, Bangladesh, found that 210 household contacts/1,000 index patients were infected with *V. cholerae* during a 21-day surveillance period (>90% of these infections occurred during the first week after the index patient sought care) (*7*,*12*). This high rate of cholera among household contacts probably results from a shared contaminated environmental source, such as water or food in the household, or secondary transmission from infected household members because of poor hygiene (*6*,*9*,*13*).

In Bangladesh, the current standard of care for cholera patients at hospital discharge is to provide oral rehydration solution (ORS) packets. No standard of care exists for household contacts of these patients despite their very high risk for cholera (*5*,*7*). The time that patients and their accompanying family members spend at a health facility during a severe diarrheal episode provides an opportunity for health providers to communicate information about water sanitation and hygiene (WASH) behavior change when perceived severity of diarrheal disease and perceived benefits of water treatment and hand washing with soap are likely to be highest (*14*). However, only a few studies have evaluated the effects of health facility–based WASH interventions, and none have evaluated the effects of these interventions in reducing enteric infections among household contacts of hospitalized diarrhea patients (*15*–*22*).

To initiate a standard of care for household contacts of cholera patients during the 1-week high-risk period after the index patient seeks care, we evaluated the efficacy of a hospital-based intervention promoting hand washing with soap and treatment of water called Cholera-Hospital-Based-Intervention-for-7-Days (CHoBI7) in Dhaka, Bangladesh, during June 2013–November 2014. We hypothesized that, in comparison with the standard message given to cholera patients at hospital discharge on ORS, CHoBI7 would significantly reduce cholera infections and increase hand washing with soap and treatment of water among highly susceptible household contacts of cholera patients.

## Methods

All study participants (household contacts and cholera index patients) provided informed consent; consent comprised adult participants (>18 years of age) signing an informed consent and/or parental consent form and children 12–17 years of age signing an assent form. If a study participant could not read, the consent form was read to him or her, and the participant then was asked to document his or her consent with an X in the presence of a witness. All study procedures were approved by the research Ethical Review Committee of icddr,b, Dhaka, and the Institutional Review Board of The Johns Hopkins Bloomberg School of Public Health (Baltimore, MD, USA).

We evaluated the efficacy of CHoBI7 by conducting a cluster randomized controlled trial in Dhaka during June 2013–November 2014. Suspected cholera patients seeking care at the icddr,b Dhaka Hospital were defined as persons with acute watery diarrhea (>3 loose stools during a 24-h period) and moderate to severe dehydration using the World Health Organization definition. These patients were screened for *V. cholerae* in their feces by using the Crystal VC Rapid Dipstick test (Span Diagnostics, Surat, India) (*23*,*24*). All dipstick-positive findings were confirmed by bacterial culture. All patients suspected to have cholera who resided within a police thana (ward) of Dhaka and were admitted to icddr,b Dhaka Hospital were screened for eligibility for the CHoBI7 trial. A cholera case was defined as a fecal bacterial culture result positive for *V. cholerae* in a suspected cholera patient. Cholera patients were excluded from the study if a household contact already was enrolled (currently or previously) or if they had received cholera vaccine, to avoid confounding from an ongoing cholera vaccine trial. 

Household contacts were defined as persons sharing the same cooking pot as the index patient for the previous 3 days. To be eligible for the study, household contacts had to plan to reside in the household of the index patient for the following week and could not have received cholera vaccine. Eligible household contacts in the hospital attending their ill family member at the time of cholera patient enrollment were invited to participate, and the household was visited to recruit household contacts within 36 hours after patient enrollment. Typically, cholera patients stayed at Dhaka Hospital for 24–48 hours before returning home. A cluster was defined as the index cholera patient and his or her household contacts. 

**Figure 1:**
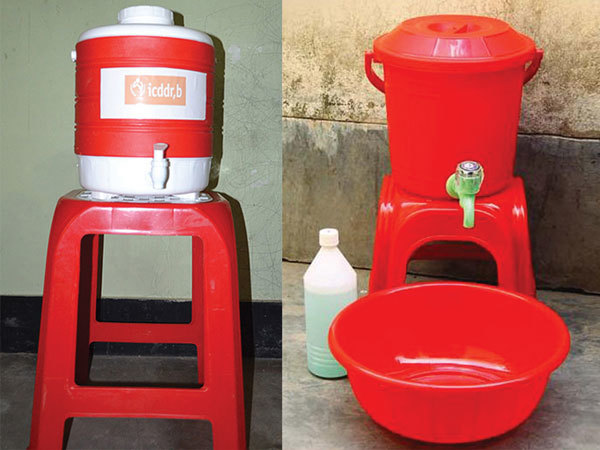
Cholera-Hospital-Based-Intervention-for-7-Days (CHoBI7) Intervention hardware, Dhaka, Bangladesh, June 2013–November 2014. The kit contained a water vessel with cover, chlorine tablets, hand washing station, and bottle of soapy water.

**Figure 2:**
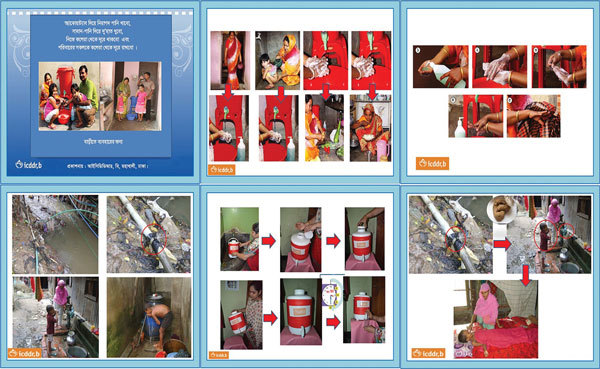
Promotional flipbook and cue cards about hand washing with soap and treatment of water, Dhaka, Bangladesh, June 2013–November 2014. Cue cards are placed next to intervention hardware as a cue to action on hygiene and water treatment–related behaviors.

The design of CHoBI7 was informed by factors from the Integrated Behavioral Model for Water, Sanitation and Hygiene interventions and constructs from the Health Belief Model (*25*,*26*). CHoBI7 was tailored to residents living in slum areas of Dhaka during 3 months of piloting and previous formative research (*27*). CHoBI7 includes 1) a pictorial (“Chobi” in Bangla) module on how cholera can spread through the environment (e.g., contamination of household drinking water sources and stored water), how persons can spread cholera to each other by contaminating food and water in their home, and instructions on proper hand washing with soap and treatment of water (Figure 1); and 2) a cholera prevention package containing a 3-month supply of chlorine tablets (Aquatabs sodium dichloroisocyanurate; Medentech, Wexford, Ireland, UK) for water treatment, soapy water bottles (a low-cost alternative to bar soap made using detergent), a hand washing station, a sealed water vessel with cover to ensure safe water storage, and cue-to-action cards with instructions about promoted behaviors (Figure 2). A trained health promoter at Dhaka Hospital delivered this pictorial module and cholera prevention package to cholera patients and their accompanying family members during a consultation session in the hospital. These messages were reinforced through daily household visits by the health promoter for the 1-week intervention period. The cost per household for CHoBI7 was US $45.50 (Technical Appendix Table 1); the cost included intervention hardware, transport cost, and the promoter’s salary.

Study recruitment at Dhaka Hospital occurred Saturday–Thursday each week during the study period. Each week, half of the surveillance days were randomly selected to be intervention days, and half were randomly assigned to be control days by using a random number generator. The principal investigator (C.M.G.) assigned randomization; this scheme limited the likelihood of seasonal variations in study arm assignment and selection bias. The control arm received the standard message given at health facilities in Bangladesh about the use of ORS to treat diarrhea, and the intervention arm received this standard message and CHoBI7. To minimize bias, we used 2 separate teams for intervention and evaluation activities.

Households were visited on days 1 (baseline), 3, 5, 7, and 9 (visits 1–5) after the cholera patient sought care at Dhaka Hospital for clinical surveillance and to assess intervention uptake indicators. For clinical surveillance, household contacts were asked whether they had diarrhea (>3 loose stools during a 24-hour period) or vomiting in the previous 48 hours, and a rectal swab sample was collected from willing household contacts at each household visit to test for *V. cholerae* in feces by bacterial culture. Because of limitations in our study personnel capacity, rectal swab samples were available only from household contacts enrolled during June 2013–June 2014.

To assess indicators of intervention fidelity, we collected a water sample from the household’s water source and drinking water stored in the home at each household visit to test for *V. cholerae* by bacterial culture and for the presence of free chlorine, as a proxy measure of water treatment, by using a digital colorimeter (Hach, Loveland, CO, USA). The US Centers for Disease Control and Prevention–recommended cutoff for free chlorine of >0.2 mg/L in household stored drinking water was used (*28*).

Spot checks were conducted at each household visit in all study households to observe whether soap was present near (within 10 steps of) the latrine and cooking areas as a proxy measure of hand washing with soap (*29*). To observe hand washing with soap practices, a 5-hour structured observation substudy was conducted once in all households recruited during October 2013–November 2014 (59 intervention and 56 control households) on surveillance day 5, 6, or 7. Hand washing with soap was recorded at the following key events promoted in CHoBI7: 1) after using the toilet, 2) after cleaning a child’s anus, 3) before eating, and 4) before preparing food.

Rectal swab samples were collected on Cary-Blair media, and water samples were collected in 500-mL bottles and transported to the Enteric Microbiology Laboratory at icddr,b. Fecal specimens and water samples were analyzed for *V. cholerae* and serotyped according to previously published methods (*30*,*31*). The laboratory was blinded to the study arm of specimens received.

Our primary outcomes were 1) the incidence of *V. cholerae*–infected household contacts, defined as a culture result positive for *V. cholerae*, and 2) the incidence of symptomatic *V. cholerae* infection, defined as diarrhea or vomiting in the past 48 hours in a *V. cholerae*–infected household contact. Our secondary outcomes were the percentages of 1) hand washing with soap at key events during 5-hour structured observation, 2) households with soap at the latrine and cooking areas, 3) households with stored drinking water with detectable *V. cholerae*, and 4) households with free chlorine concentrations >0.2 mg/L in stored drinking water. We excluded the baseline household visit from analyses of the intervention efficacy because the intervention had not yet been provided to household members. The cost per cholera case and case averted was calculated by using the assumptions in Technical Appendix Table 2.

We used Optimal Design software (University of Michigan, Ann Arbor, MI, USA) for the sample size calculation to determine the number of cholera cases (clusters of household contacts) needed to reject the null hypothesis that the incidence of cholera did not significantly differ by study arm at a 95% CI and 80% power (*32*). We assumed that cholera infection would occur in 20% of household contacts in the control arm and that the intervention would reduce this rate to 10% with an average cluster size of 3 household contacts (*7*). On the basis of these assumptions, we estimated needing 156 index cholera patients and 468 household contacts (78 cholera patients and 234 household contacts in each study arm).

To compare baseline household- and individual-level characteristics by study arm, we conducted a χ^2^ test for categorical variables, a 2-sample *t* test for continuous variables, and a Fisher exact test when <5 values were in a category. Logistic regression models were performed to estimate the odds of developing cholera and to compare intervention uptake indicators during visits 2–5 by study arm using generalized estimating equations to account for clustering within households and approximate the 95% CI. These analyses were performed by using SAS version 9.3 (SAS Institute Inc., Cary, NC, USA). For study variables where 1 study arm had no events, we used Fisher exact tests at the household level to determine a significant difference between study arms. To calculate exact 95% CIs in this instance, we used an algorithm to invert test statistics in R (R Core Team, Vienna, Austria) (*33*).

## Results

**Figure 3:**
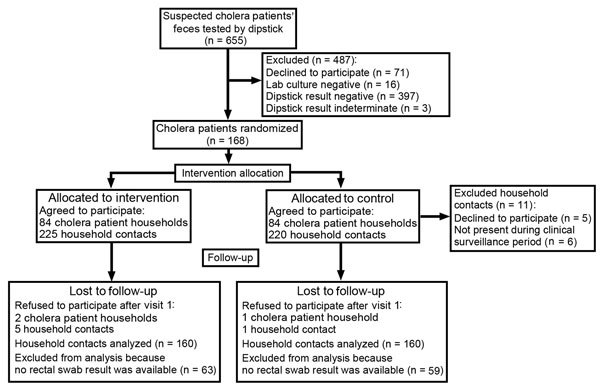
Flowchart of study participation in randomized controlled trial of cholera hospital-based intervention for 7 days, Dhaka, Bangladesh, June 2013–November 2014.

Of the 655 suspected cholera cases screened by Crystal VC Rapid Dipstick for the presence of *V. cholerae* in feces, 255 (39%) were dipstick positive; 400 (61%) results were negative or indeterminate (Figure 3). Of persons with dipstick-positive samples, 71 declined to participate, and 16 were negative for *V. cholerae* by bacterial culture.

We invited all eligible household members in study households during the baseline surveillance visit to participate in the trial. Of household members in cholera patient households, 27% (229/853) were unavailable for the baseline interview and therefore were not enrolled in the trial. The proportion of household members available for the baseline interview did not differ significantly by study arm (26% intervention arm vs. 29% control arm, p = 0.45). Of the 453 household contacts screened for eligibility, 5 declined to participate, and 6 were not home during the clinical surveillance period.

Two intervention and 1 control household refused to participate after the baseline visit. Therefore, 84 cholera patients and 225 household contacts were allocated to the intervention arm and 84 cholera patients and 220 household contacts to the control arm. Baseline index patient, household contact, and household characteristics did not differ significantly by study arm (Table 1). During the study period, 27% of control households had at least 1 water source (e.g., water pump) sample that tested positive for *V. cholerae* compared with 33% of intervention households (p = 0.4).

**Table 1:** Demographic and environmental characteristics of households of patients with cholera, by study arm, Dhaka, Bangladesh, June 2013–November 2014*

Characteristic	Control arm	Intervention arm	p value†
No. households	83	82	
No. enrolled household contacts, median ± SD (min–max)	2 ± 0.9 (2–6), n = 220	2 ± 0.8 (2–5), n = 219	0.9
Index patient			
Female sex, no. (%)	56 (67)	52 (63)	0.5
Age, y, median ± SD (min–max)	25 ± 17.6 (0.67–95)	25 ± 15 (1–65)	0.3
<5, no. (%)	5 (6)	8 (10)	0.6
5–14, no. (%)	16 (19)	17 (21)	
>14, no. (%)	62 (75)	57 (70)	
Household contact‡			
Female sex, no. (%)	135 (61)	126 (58)	0.3
Age, y, median ± SD (min–max)‡	13 ± 15 (0.75–67), n = 220	13 ± 16 (0.58–75), n = 219	0.3
<5 years, no. (%)	36 (16)	45 (21)	–
5–14, no. (%)	84 (38)	68 (31)	0.3
>14, no. (%)	103 (46)	106 (48)	0.4
Television ownership, no. (%)	42 (51)	45 (55)	0.5
Electricity, no. (%)	82 (99)	82 (100)	0.3
Refrigerator ownership, no. (%)	12 (14)	9 (11)	0.5
A household member can read and write, no. (%)	67 (81)	72 (88)	0.2
Educational level of person responsible for primary drinking water collection, no. (%)			
No formal education	40 (48)	40 (49)	0.3
Primary school	31 (37)	24 (29)	
Secondary school	11 (13)	17 (21)	
Higher secondary school	0	0	
Bachelor’s degree	1 (1)		
Master's degree	0	1 (1)	
Water source type, no. (%)			
Groundwater	45 (54)	46 (46)	0.3
Piped water supply	380 (46)	34 (41)	
Baseline presence, no. (%)			
Any type of soap in latrine area of household	13 (16)	9 (11)	0.3
Any type of soap in cooking area of household	10 (12)	8 (10)	0.6
*Vibrio cholerae* in stored drinking water	5 (6)	9 (11)	0.2
*V. cholerae* in source water	10 (12)	12 (15)	0.6
Presence of *V. cholerae* in source water during study period	22 (27)	27 (33)	0.4

*Unless otherwise specified, the denominator for the control are is 83 and for the intervention arm 82. †χ^2^ test for categorical variables and 2-sample *t* test for continuous variables. ‡p values were calculated by using generalized estimating equations to account for the clustering of the data at the household level.

Culture results for *V. cholerae* were available for 320 (73%) household contacts. Enrolled household contacts with or without rectal swab culture results available did not differ significantly in clinical or demographic characteristics (online Technical Appendix Table 3).

**Table 3:** Odds ratios for hand washing with soap and water treatment and indicators of water quality in an intervention study of *Vibrio cholerae*, Dhaka, Bangladesh, June 2013–November 2014

Outcome	No. complying/no. persons (%)		Odds ratio* (95% CI)	p value*
	Control arm	Intervention arm		
Hand washing with soap events at key times during 5-h structured observation	50/629 (8)	418/759 (55)	14.68 (8.32–25.90)	<0.0001
Hand washing with soap events after toileting during 5-h structured observation	23/123 (19)	144/197 (73)	12.14 (5.68–25.93)	<0.0001
Household visits with soap in latrine area, visits 2–5†	50/332 (15)	326/327 (99.7)	1,842.36 (241.53–145,054.53)	<0.0001
Household visits with soap in kitchen area, visits 2–5†	43/332 (13)	317/327 (97)	213.64 (62.59–729.24)	<0.0001
Households visits with detectable free chlorine >0.2 mg/L in household stored drinking water, visits 2–5‡	1/332 (<1)	308/327 (94)	4,878.62 (799.30–4.503 × 10^15^)	<0.0001
Household visit with stored water with detectable *V. cholerae*, visits 2–5	5/83 (6)	0/82 (0)	0.00 (0–1.08)§	0.06¶
Household visit with source water with detectable *V. cholerae*, visits 2–5	15/83 (18)	22/82 (27)	1.66 (0.79–3.49)	0.18

*Logistic regression using generalized estimating equations. †Soap present within 10 steps of the latrine or cooking area at household visits during the intervention period. ‡Cutoff recommended by the Centers for Disease Control and Prevention (Atlanta, GA, USA). §To calculate exact 95% CIs, an algorithm was used to invert test statistics. ¶Fisher exact test.

A total of 148 (93%) control and 140 (88%) intervention household contacts were negative for *V. cholerae* at the baseline visit (p = 0.30) (Table 2). Intervention contacts had a 47% lower incidence of *V. cholerae* infection (symptomatic and asymptomatic) than control contacts (odds ratio [OR] 0.50, 95% CI 0.21–1.18) during the intervention period. Furthermore, intervention contacts had no symptomatic *V. cholerae* infections, compared with 5% of control contacts (OR 0.00, 95% CI 0–0.623). On the basis of these findings, we determined the cost per cholera case (symptomatic *V. cholerae* infection) averted would be US $227.50 ($227.50–$598.68) (Technical Appendix Table 2). We calculated the range for the cost estimate using the 95% CI for the OR of a symptomatic *V. cholerae* infection.

**Table 2:** Evaluation of intervention efficacy to reduce *Vibrio cholerae* infection among household contacts of cholera patients during the intervention period (visits 2–5), Dhaka, Bangladesh, June 2013–November 2014

Household contact characteristic	No. (%) contacts		Odds ratio (95% CI)	p value*
	Control arm	Intervention arm		
Culture results available	160 (100)	160 (100)	–	–
Negative for *V. cholerae* infection at baseline	148 (93)	140 (88)	1.15 (0.88–1.51)	0.30
Initial *V. cholerae* infections during the intervention period	20 (14)	10 (7)	0.50 (0.21–1.18)	0.11
Initial symptomatic *V. cholerae* infections during intervention period†	8 (5)	0	0.00 (0–0.623)‡	0.006§

*Calculated with logistic regression model by using generalized estimating equations to account for clustering within study households. †Symptomatic infection defined as a *V. cholerae*–infected household contact with diarrhea or vomiting in the past 48 hours. ‡To calculate exact 95% CIs, an algorithm was used to invert household-level test statistics. §Fisher exact test calculated at the household level.

The odds of hand washing with soap at key events during the structured observation period were 14 times higher in the intervention arm than in the control arm (OR 14.68, 95% CI 8.32–25.90) (Table 3), and the odds of hand washing with soap after toileting were 12 times higher in the intervention arm than in the control arm (OR 12.14, 95% CI 5.68–25.93). A significantly higher proportion of household visits in the intervention arm than in the control arm had soap present at the cooking area (99.7% vs. 15%, p<0.0001) and latrine area (98% vs. 13%, p<0.0001) during the intervention period (Table 3). *V.* *cholerae* was present in no stored drinking water samples in households in the intervention arm and in 6% of samples in the control arm during the intervention period (OR 0, 95% CI 0–1.08). The proportion of households with free chlorine concentrations >0.2 mg/L was significantly higher in the intervention arm than in the control arm (94% vs. <1%, p<0.0001).

## Discussion

CHoBI7 significantly reduced symptomatic *V. cholerae* infections and reduced overall *V. cholerae* infections by nearly half during the intervention period. Consistent with these findings, the odds of hand washing with soap at key events during the structured observation were 14 times higher in the intervention arm than in the control arm, and nearly all intervention households had free chlorine concentrations in stored drinking water in the Centers for Disease Control and Prevention–recommended range. In addition, no stored drinking water samples in intervention households had detectable *V. cholerae*. These findings demonstrate that CHoBI7 was highly effective in reducing symptomatic cholera and increasing hand washing with soap and treatment of water during the 1-week high-risk period for household contacts of cholera patients.

We attribute the success of the CHoBI7 intervention to several key factors. First, this intervention was delivered during a time of severe illness in these households, when perceived severity of diarrheal disease and perceived benefits of hand washing with soap and treatment of water were likely to be high. Previous studies have found that during outbreaks of severe disease, such as cholera, households have higher perceived severity of diarrheal disease and greater perceived benefits of water treatment (*14*,*34*,*35*). Consistent with this observation, in Dhaka in 2013, use of a community-level point-of-use chlorine dispenser peaked after cholera-associated deaths in a slum area (L. Unicomb, pers. comm.). Second, we provided hardware that was pretested in a pilot study and facilitated the promoted behaviors (hand washing with soap and treatment of water) (*27*). Third, we trained health promoters to reinforce the promoted behaviors by using the CHoBI7 pictorial module, which probably led to a favorable environment for habit formation (*25*).

CHoBI7 significantly reduced symptomatic *V. cholerae* infection but not overall infection. We suspect the reason is our small sample size and the intervention reducing the infecting inoculum size within households to a level below which symptomatic infections could occur. Consistent with this hypothesis, a previous challenge trial found that symptomatic infections could occur at an inoculum size of 10^4^ CFUs of *V. cholerae* and that illness severity was based on the size of the infecting inoculum (*36*).

Major advantages of the CHoBI7 intervention are its focus on high-risk persons during the 1-week period when they are most susceptible to cholera infections and its dissemination in a clinical setting in which cholera cases can be rapidly identified by dipstick test. Furthermore, the intervention is relatively inexpensive (US $227.50/cholera case averted) and would be likely to be more cost effective than a similar WASH intervention implemented as a community-based intervention, given the much lower prevalence of cholera in the general population (1.6 cholera cases/1,000 general population vs. 50 cholera cases/1,000 household contacts of cholera patients) (*12*,*37*). A recent study that used a cholera vaccine cost-effectiveness calculator found that a cholera vaccination program targeting geographic hotspots for cholera (cholera incidence >10 cases/1,000 year) in Bangladesh would cost US $226 per cholera case averted, similar to the cost of CHoBI7 (*38*).

To our knowledge, only 1 intervention study has been published that evaluated the effectiveness of safe water storage and water treatment on cholera transmission among household contacts of cholera patients. This study, conducted in Calcutta, India, resulted in a 59% reduction in overall cholera infections in the chlorine water treatment arm and a 76% reduction in the narrow-necked water pitcher arm during the 5-day intervention period (*39*). An earlier intervention study in Dhaka found that promotion of hand washing with soap among household contacts of shigellosis patients resulted in an 85% reduction in symptomatic *Shigella* infections during the 10-day intervention period (*40*). These findings are consistent with those from our trial and suggest that WASH interventions directed toward the high-risk period for household contacts of hospitalized diarrhea patients might be a promising approach for reducing transmission of enteric pathogens in this susceptible population.

Our study has a few limitations. First, because CHoBI7 combined hand washing with soap and treatment of water, we cannot establish the effect of these interventions individually. Second, our sample size was small, and we were unable to obtain culture results from as many household contacts as anticipated because of limited study personnel capacity. This limitation reduced our power to detect a significant difference in primary outcome between the 2 study arms (80% vs. 69%). Third, 27% of household members of cholera patients were not present during the baseline surveillance visit and therefore were not enrolled as household contacts. These persons are likely to have been the household members who spent the most time outside the home during the study period. However, the proportion of household members who were unavailable for the baseline interview did not differ significantly by study arm. Fourth, the study could not be unblinded; however, to minimize potential bias, the evaluation and intervention teams were separate, and the laboratory was blinded to intervention assignment. Fifth, this study was an efficacy trial. Our objective was to evaluate whether hand washing with soap at key events and treatment of household stored drinking water consistently would significantly reduce *V. cholerae* infections. Future studies should conduct an effectiveness trial to identify whether a hospital-based intervention only (without home visits) can lead to sustained uptake of the promoted hand washing with soap and treatment of water during the 1-week high-risk period for these households.

In our study, CHoBI7 significantly reduced symptomatic *V. cholerae* infections among household contacts of cholera patients in urban Dhaka, Bangladesh. These findings suggest that this hospital-based intervention is a promising, cost-effective approach that could be initiated as a standard of care for household members of cholera patients. Future studies should investigate the efficacy of CHoBI7 in other settings affected by cholera globally, evaluate the effects of CHoBI7 on other enteric pathogens, and identify effective low-cost approaches to take CHoBI7 to a larger scale.

Technical Appendix Cost per household of Cholera-Hospital-Based-Intervention-for-7-Days (CHoBI7), Dhaka, Bangladesh, June 2013–November 2014; calculation of cost per *Vibrio cholera*e infection and cholera case averted for CHoBI7; and clinical and demographic characteristics by study arm of study participants with rectal swab results available.
