# Supplementary material for: Randomized Controlled Trial of Hospital-Based Hygiene and Water Treatment Intervention (CHoBI7) to Reduce Cholera
Source: Emerg Infect Dis. 2016 Feb;22(2):233–41. doi: 10.3201/eid2202.151175 (PMC4734520; doi:10.3201/eid2202.151175)
Supplement: Technical Appendix — Cost per household of Cholera-Hospital-Based-Intervention-for-7-Days (CHoBI7), Dhaka, Bangladesh, June 2013–November 2014; calculation of cost per Vibrio cholerae infection and cholera case averted for CHoBI7; and clinical and demographic characteristics by study arm of study participants with rectal swab results available. [file 15-1175-Techapp-s1.pdf]

Article DOI: http://dx.doi.org/10.3202/eid2201.151175

Randomized Controlled Trial of Hospital-Based Hygiene and Water Treatment Intervention (CHoBI7) to Reduce Cholera

Technical Appendix

Technical Appendix Table 1. Cost per household of Cholera-Hospital-Based-Intervention-for-7-Days intervention, Dhaka, Bangladesh, June 2013–November 2014†

Item	Rate/unit*
Health promoter salary, US $120/mo†	12.00
Promoter transportation for 7 household visits	9.00
Hand washing station	12.00
12-L covered drinking water vessel	9.00
Detergent for soapy water for 1 wk	0.30
Promoter communication materials	3.00
Water treatment tablets for 1 wk	0.20
Cost for intervention/household, US $†	45.50

*These estimates assume that 1 promoter can serve 10 households/mo.
†Calculated by dividing the monthly salary per promoter by the 10 households they can serve each month.

Technical Appendix Table 2. Calculation of cost per *Vibrio cholerae* infection and cholera case averted for CHoBI7, Dhaka, Bangladesh, June 2013–November 2014*

	Cost per symptomatic case of *Vibrio cholerae* infection averted	
Characteristic	Value	Calculation
Cholera cases in households **without** CHoBI7, nonbaseline	0.20 Cholera cases/household	Without the intervention, expected cholera cases in households during visits 2–5 would be: as follows 5% (cholera case from control arm visits 2–5) × 4 (susceptible household contacts) = 0.20, assuming 4 susceptible household contacts/household during visits 2–5
Cholera cases in households **with** CHoBI7, nonbaseline	0 Cholera cases/household	With the intervention, expected cholera cases in household during visits 2–5 would be as follows: (100%–100% [CHoBI7 efficacy]) × 5% (cholera cases from control arm visits 2–5) × 4 (susceptible household contacts) = 0
Cholera cases averted in households with CHoBI7	0.20 Cholera cases/household	By using these assumptions the intervention would avert: 0.20 (cholera cases without CHoBI7) −0.0 (cholera cases in households with CHoBI7) = 0.20 cholera cases per household
Cost/cholera case averted	US $227.50/cholera case averted	Cost per cholera case averted would be as follows: US $45.50 (cost of the CHoBI7)/0.20 (cholera cases averted) = US $227.50. A range of US $227.50 USD–US $598.68 was added by using the 95% CI for the odds ratio of a symptomatic *V. cholerae* infection.

*CHoBI7, cholera hospital-based intervention for 7 days.

Technical Appendix Table 2. Clinical and demographic characteristics by study arm by study participants with rectal swab results available, Dhaka, Bangladesh, June 2013–November 2014

Characteristic	Yes*	No*	p value†
No. enrolled household contacts	320 (73)	122 (27)	
Intervention arm	160 (49)	60 (50)	0.65
Sex, F	193 (60)	68 (56)	0.52
Mean age of household contact ± SD, y (min-max)	18 ± 15.3 (0.7–75), n = 320	20 ± 16.4 (0.6–72), n = 122	0.12
Diarrhea during surveillance period	103 (33)	40 (32)	0.73
Vomiting during surveillance period	33 (9)	11 (10)	0.68

*All values are no. (%) unless otherwise indicated.
†p values calculated by using generalized estimating equations to account for the clustering of data at the household level.
